# Determinants of postnatal care use at health facilities in rural Tanzania: multilevel analysis of a household survey

**DOI:** 10.1186/s12884-015-0717-7

**Published:** 2015-10-30

**Authors:** Diwakar Mohan, Shivam Gupta, Amnesty LeFevre, Eva Bazant, Japhet Killewo, Abdullah H Baqui

**Affiliations:** Department of International health, Johns Hopkins Bloomberg School of Public Health, Baltimore, Maryland; Jhpiego, Baltimore, Maryland; Department of Biostatistics & Epidemiology, Muhimbili University of Allied Health Sciences, Dar es Salaam, Tanzania

**Keywords:** Postnatal care, multilevel model, Tanzania

## Abstract

**Background:**

Postnatal care (PNC) for the mother and infant is a neglected area, even for women who give birth in a health facility. Currently, there is very little evidence on the determinants of use of postnatal care from health facilities in Tanzania.

**Methods:**

This study examined the role of individual and community-level variables on the use of postnatal health services, defined as a check up from a heath facility within 42 days of delivery, using multilevel logistic regression analysis. We analyzed data of 1931 women, who had delivered in the preceding 2–14 months, from a two-stage household survey in 4 rural districts of Morogoro region, Tanzania. Individual level explanatory variables included i) Socio-demographic factors: age, birth order, education, and wealth, ii) Factors related to pregnancy: frequency of antenatal visits, history of complications, mode of delivery, place of delivery care, and counseling received. Community level variables included community levels of family planning, health service utilization, trust, poverty and education, and distance to health facility.

**Results:**

Less than one in four women in Morogoro reported having visited a health facility for postnatal care. Individual-level attributes positively associated with postnatal care use were women’s education of primary level or higher [Odds Ratio (OR) 1.37, 95 % Confidence Interval (CI) 1.04–1.81], having had a caesarean section or forceps delivery (2.95, 1.8–4.81), and being counseled by a community health worker to go for postnatal care at a health facility (2.3, 1.36–3.89). Other positive associations included those recommended HIV testing for baby (1.94, 1.19–3.15), and whose partners tested for HIV (1.41, 1.07–1.86). High community levels of postpartum family planning usage (2.48, 1.15–5.37) and high level of trust in health system (1.77, 1.12–2.79) were two significant community-level predictors. Lower postnatal care use was associated with having delivered at a hospital (0.5, 0.33–0.76), health center (0.57, 0.38–0.85), or dispensary (0.48, 0.33–0.69), and having had severe swelling of face and legs during pregnancy (0.65, 0.43–0.97).

**Conclusions:**

In the context of low postnatal care use in a rural setting, programs should direct efforts towards reaching women who do not avail themselves of postnatal care as identified in our study.

## Background

Globally an estimated 254,700 women died in 2010 from complications associated with pregnancy or childbirth [1]. Almost all (99 %) of these deaths occurred in low resource settings; more than half of these occurring in Sub-Saharan Africa [2]. Among the 7.6 million deaths in children under the age of 5, 44 % occur during the neonatal period and nearly half of these deaths occur during the first 72 hours following delivery [3]. Tanzania experiences high levels of maternal mortality (410 per 100,000 live births) and under-five child mortality (81 per 1000 live births) with 32 % of the latter mortality occurring in the first month of life [4].

**Fig. 1:**
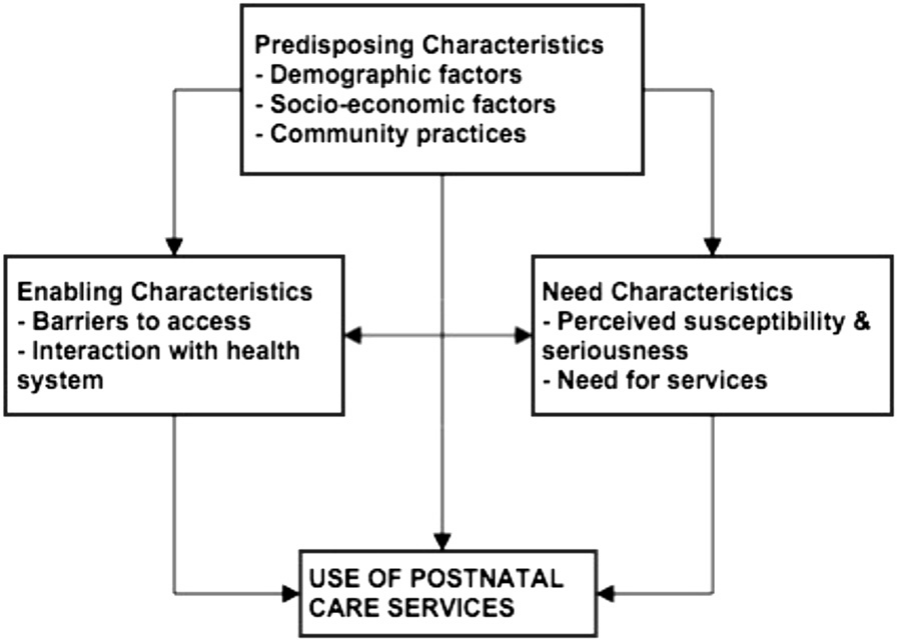
Factors influencing utilization of postnatal care

Evidence suggests that integrated community-based services provided from pre-pregnancy through the postnatal period can improve maternal and neonatal outcomes, though the evidence from Africa has been conflicting [5–12]. The ‘continuum of care’ approach (Fig. 1) has now been highlighted as a core programmatic principle of improving Maternal Newborn & Child Health (MNCH) [13–15]. This framework strives to provide all women and children with access to care during pregnancy, childbirth and the postnatal period by integrating effective interventions and delivery strategies within existing health system packages [14].

Across the continuum of care, significant disruptions occur in the continuity of services from the antenatal period to the postnatal period. For example, 95 % of pregnant women in Tanzania make at least one antenatal visit while 50 % deliver with a skilled birth attendant, yet only 35 % of Tanzanian women receive a postnatal checkup [4]. Postnatal care (PNC) for the mother and infant in the crucial first hours and days after childbirth is poor in quality and coverage or missing entirely, even for women who give birth in a health facility [16]. According to the World Health Organization (WHO), the postnatal period starts one hour post-delivery of the placenta and continues for six weeks thereafter. Postnatal care includes the prevention of complications, their early detection and treatment, and provision of services on breastfeeding, birth spacing, immunizations, nutrition and HIV. Immediate postnatal care can help reduce mortality and morbidity for the mother and newborn, which are very high during the first week and up to four weeks after delivery (Koblinsky MA. Community-based postpartum care: an urgent unmet need. Unpublished 2005). The postnatal care visit also serves as an entry point for the provision of other health services including family planning and immunization.

Although PNC includes services provided to women and newborns both in the community and health facility, the focus of the present study is to explore the issue of postnatal visits at health facilities for mothers in Tanzania. Our research is on the maternal aspect of the continuum of care framework, and hence focused on the points of contact for postnatal care for the mother. Since pre-discharge care after childbirth is delivered as part of the ‘delivery care’ contact, we have excluded care received at discharge after delivery care from our analysis. The Tanzanian Ministry of Health and Social Welfare (MoHSW) guidelines call for at least 3 visits during the postnatal period (within one week, at 28 days and at 42 days) apart from the immediate postnatal care around the time of delivery [17]. CHWs are expected to encourage the mother to attend postnatal appointments at the health facility according to the following schedule – i) Within 48 h in case of home deliveries, ii) Within seven days, iii) At 28 days, iv) At 42 days, v) At six months. These visits (with the exception of the 48 h visit for home deliveries) are expected to be made by women, irrespective of whether they deliver at a facility or at home. There is a need to understand the differences in the characteristics of the individuals and their communities who do not use these services. The objective of this study is to examine factors that are associated with the use of postnatal care services in rural Morogoro, Tanzania.

## Methods

### Study design and context

The target population for our research was rural women who had a childbirth in the preceding 2–14 months and referred to as “recently delivered women” (RDW). The household survey of women (*N* = 1968) was carried out from August 2011 to November 2011. The baseline household survey was conducted as part of an evaluation of the Integrated Facility and Community (IFC) program being implemented by the MoHSW with support from Jhpiego since 2008. The principal objective of the survey was to establish baseline household characteristics and MNCH care utilization among RDWs.

### Study area and sampling

The study sample for the survey was RDWs living in rural areas of four districts in the Morogoro Region of Tanzania- Morogoro District Council, Mvomero, Kilosa and Ulanga. We performed post-hoc power analysis for our sample size of 1931 women - among the respondents, 37 (2 %) were excluded from the analysis because they had experienced pregnancies that resulted in a miscarriage and did not have reason to access postnatal care. The sample size would have 80 % power to detect a 10 % difference in the proportion of women using postnatal care between two groups with a type 1 error of 0.05, assuming an intra class correlation (ICC) of 0.1. A two-stage sampling strategy was employed to select households from 60 clusters, − a ‘cluster’ being defined as a unit of 1000 population. The first stage was the selection of villages (containing the cluster) through probability proportional to size (PPS) sampling using population estimates from the 2002 Tanzania National Population Census. In each selected village, a list of the population and households was made for the constituent sub-village units (Vitongoji) based on data from the local government authorities which were then grouped to create clusters composed roughly of a population of 1000. The second stage of the sampling process was to choose one cluster in a random manner by lottery. In each cluster, the survey team visited every household to list and interview women who had any pregnancy outcome (live born/stillbirth/abortion) in the preceding 2–14 months. The probability of selection for each household in the sample was equal. If the household had more than one eligible woman, only one was randomly selected for interview.

### Instrument and data collection

The respondent identified in each household was interviewed using a questionnaire adapted from the model questionnaires developed by the MEASURE Demographic & Health Surveys (DHS) program [18]. The questionnaire was adapted to reflect relevant issues related to the larger ongoing evaluation in the region and collect information on background characteristics, pregnancy history, utilization of health care during pregnancy, delivery, and postnatal period as well as barriers to care seeking. If multiple births were encountered, then it was considered as a single pregnancy event. The adapted questionnaire was translated from English into Kiswahili and pilot tested in a district adjacent to the study site (Pwani Region). The respondents were interviewed after obtaining written informed consent. Two teams of trained interviewers fluent in Swahili and English administered the survey questionnaire. A field editor reviewed questionnaires from all the teams using a checklist for completeness, quality, and consistency at the end of each day while the study investigators made periodic checks to ensure quality of data collection and entry.

### Variables

We framed the use of postnatal care adapting the healthcare-seeking behavior model developed by Anderson and Newman [19]. This model proposes that the use of health care services is a function of three sets of characteristics - predisposing characteristics, enabling characteristics, and need characteristics (Fig. 1). We included (i) predisposing characteristics such as age, parity, marital status, education, wealth index, community poverty and peer usage of services, (ii) enabling characteristics such as distance to facilities, cost of services, trust in health system and community outreach activities, and (iii) need characteristics such as perceived susceptibility, seriousness of complications during antenatal, delivery and postnatal period, mode of delivery and need for contraception, and HIV testing.

The outcome variable was ‘use of postnatal care’, which was defined as attending postnatal care for the mother's care at a dispensary, health center, or hospital (government or private) within 6 weeks of delivery. Individual-level explanatory variables included demographic variables such as woman’s age, birth order, education, marital status, and religion. An index of household wealth, based on household assets was created using principal components analysis (PCA) methods proposed by Filmer and Pritchett and used to group households into wealth quintiles [20]. Frequency of antenatal care visits and location of delivery care were included as measures of health system utilization. The variable ‘HIV testing of baby’ refers to the requirement for HIV counseling and testing of the baby during a postnatal visit and functions as a ‘need characteristic’ variable. The ‘partner test for HIV’ variable is proxy for the involvement of men in the process. If expenses were incurred for a delivery, it was coded as a dichotomous variable ‘money spent on delivery’ which is a proxy barrier to care seeking.

To create community-level variables, the individual level responses were averaged at the level of the clusters. The asset score of the households in the cluster was averaged to generate a community poverty score that served as proxy for wealth of the community. The proportion of women in the cluster with primary or higher education was used as proxy for literacy of a community. The proportion of women using contraception and the proportion attending 4 or more antenatal visits were used as proxies for community family planning practices and maternal health service utilization. The community level variables for education, contraceptive prevalence and ANC4+ coverage were generated by assigning each cluster with the value of the prevalence of women for the indicators and dividing them into low, middle and high categories. Communities with more than 80 % of respondents who reported trusting a health provider or CHW for advice on pregnancy related issues were classified as communities with high trust. Survey teams, also, collected information on the presence of the nearest functioning health facility and the distance recorded. The distance variable was categorized as 0 km (facility in the village), less than 5 km and more than 5 km.

### Statistical analysis

Frequency distributions of the sample women were explored to describe the characteristics of women included in this study. Bivariate and multivariable analyses were performed for individual-level and community-level variables of interest. Multilevel models take into account the hierarchical structure of the data and clustering of responses at the different levels. The following equation illustrates the multilevel model for utilization of Postnatal Care

$$ logit\left({P}_{ij}\right)=b0+b1{I}_{ij}+b2{C}_j+b3{Z}_j{X}_{ij}+{\upmu}_j+{\upvarepsilon}_{ij} $$

where i and j are the level 1 (individual) and level 2 (community) units respectively; p_ij_ is the probability of the outcome of interest for woman *i* in the cluster *j*; the b’s are the fixed coefficients; I and C refer to individual-level and community-level explanatory variables, respectively; and Z_j_X_ij_ is a cross-level interaction term; μ shows the random effects for the jth cluster. The error term, ε, represents unmeasured factors that may influence use of postnatal care at a health facility. A multilevel random intercept logistic regression model without covariates (null model) was used to assess the influence of unobserved community-level characteristics on the overall variation in facility use. Three multilevel random intercept logistic regression models were fitted to estimate associations between the individual and community variables and the likelihood of seeking postnatal care at a health facility. The first model included individual-level characteristics only, the second model included community-level characteristics only and the last model (full) includes individual and community-level variables. The independent variables were retained in each of the models if the the *p*-value was less than 0.2. Important demographic variables were also retained in the multivariable models, in addition to the variables chosen from the bivariate analysis, based on previous literature on the use of maternal health services. All statistical analyses were carried out with STATA 13.1[21].

The extent of missing data was assessed, and patterns of missingness were explored in order to ensure whether data were missing at random. Approximately 15% of our sample was missing data for at least one variable in the final multilevel model. We used multivariate imputation using chained equations (MICE), which uses a Gibbs-like algorithm to impute multiple variables sequentially using univariate fully conditional specifications. This method is considered more appropriate for the imputation of categorical data because it does not assume normality of imputed variables [22]. Independent variables with more than 2% of values missing were imputed, and 50 concatenated datasets were created to reduce any potential bias caused by rounding. Variables imputed included the outcome variable of use of postnatal care, complications during pregnancy, delivery and postnatal period, counseling from CHW regarding postnatal care, mode of delivery, money spent on delivery, partner test for HIV and HIV testing of baby. The full multilevel model was subjected to sensitivity tests to estimate the impact of missing data using multivariate imputations for the independent variables. The percentage change in the standard error for all independent variables was less than 0.5 % and the estimates derived from the imputed model are used.

### Ethical considerations

Ethical and administrative approvals were obtained from the Ethics Review Committee of the Johns Hopkins University and the Muhimbili University of Allied Health Sciences, Dar es Salaam, Tanzania. Written informed consent was obtained from each participant.

## Results

### Population characteristics and study population

Our study included 1931 women who gave birth at any location residing in 60 clusters in four districts of Morogoro region in Tanzania. Almost two thirds of the population were Christian while 81% reported living within a marriage or a union. About 98 % had made 1or more antenatal care visits and 34 % reported having delivered at home. Thirty-one percent reported having experienced a complication during the antenatal period and 14 % and 11 % reported having complications during delivery and in the postnatal period respectively, with 6 % reporting a complicated mode of delivery (caesarean section or use of forceps). Less than a quarter of women (437, 23.2 % 95 % CI: 18.7–28.3) of the respondents reported visiting a health facility to receive postnatal care with 2.4 % (1.6–3.4) receiving care from a hospital, 9.3 % (5.5–15.2) from a health center and 10.8 % (8.2–14.2) from a dispensary. Some key characteristics of women associated with the type of facility accessed for postnatal care is given in Table 1.

**Table 1:** Distribution of selected socio-demographic characteristics and postnatal care use across different types of facilities

	Type of facility for PNC						No postnatal care (*n* = 1451)	
	Hospital (*n* = 44)		Health Center (*n* = 174)		Dispensary (*n* = 203)			
	Col %	95 % CI	Col %	95 % CI	Col %	95 % CI	Col %	95 % CI
Age								
15–19	20.5 %	[10.6 %–35.8 %]	14.5 %	[9.9 %–20.6 %]	12.9 %	[9.1 %–17.8 %]	15.4 %	[13.5 %–17.5 %]
20–34	61.4 %	[44.7 %–75.7 %]	69.9 %	[63.1 %–76.0 %]	72.3 %	[67.1 %–76.9 %]	69.4 %	[66.4 %–72.2 %]
35–49	18.2 %	[10.6 %–29.4 %]	15.6 %	[11.4 %–21.0 %]	14.9 %	[10.4 %–20.7 %]	15.2 %	[12.9 %–17.8 %]
Birth order								
First pregnancy	27.3 %	[15.4 %–43.6 %]	20.9 %	[15.8 %–27.2 %]	19.2 %	[14.4 %–25.2 %]	21.6 %	[19.4 %–24.0 %]
Second or higher	72.7 %	[56.4 %–84.6 %]	79.1 %	[72.8 %–84.2 %]	80.8 %	[74.8 %–85.6 %]	78.4 %	[76.0 %–80.6 %]
Education								
Less than primary	29.5 %	[18.9 %–43.1 %]	28.3 %	[19.7 %–38.8 %]	35.5 %	[28.4 %–43.3 %]	39.8 %	[35.7 %–43.9 %]
Primary completed or higher	70.5 %	[56.9 %–81.1 %]	71.7 %	[61.2 %–80.3 %]	64.5 %	[56.7 %–71.6 %]	60.2 %	[56.1 %–64.3 %]
Wealth								
Lowest	20.5 %	[10.8 %–35.3 %]	13.8 %	[7.2 %–24.8 %]	27.6 %	[19.9 %–36.9 %]	22.3 %	[18.1 %–27.0 %]
Second	18.2 %	[9.6 %–31.7 %]	19.0 %	[10.8 %–31.1 %]	15.8 %	[10.4 %–23.2 %]	18.7 %	[16.0 %–21.8 %]
Middle	18.2 %	[9.8 %–31.3 %]	20.7 %	[13.4 %–30.5 %]	19.7 %	[14.7 %–25.9 %]	20.1 %	[17.4 %–23.0 %]
Fourth	13.6 %	[5.4 %–30.4 %]	21.8 %	[12.9 %–34.5 %]	20.2 %	[14.6 %–27.3 %]	19.7 %	[16.9 %–22.9 %]
Highest	29.5 %	[17.5 %–45.3 %]	24.7 %	[15.1 %–37.8 %]	16.7 %	[11.1 %–24.6 %]	19.2 %	[14.7 %–24.8 %]
Place of Delivery								
Hospital	77.3 %	[56.9 %–89.7 %]	10.9 %	[6.3 %–18.3 %]	11.4 %	[7.1 %–17.7 %]	20.1 %	[16.8 %–23.8 %]
Health Center	6.8 %	[2.2 %–19.1 %]	46.6 %	[27.5 %–66.6 %]	10.4 %	[6.1 %–17.1 %]	20.1 %	[15.5 %–25.6 %]
Dispensary	2.3 %	[0.3 %–15.1 %]	14.4 %	[5.4 %–32.8 %]	30.2 %	[20.7 %–41.7 %]	26.4 %	[21.8 %–31.7 %]
No delivery care	13.6 %	[5.7 %–29.3 %]	28.2 %	[13.8 %–49.0 %]	48.0 %	[36.4 %–59.8 %]	33.4 %	[26.4 %–41.3 %]
At least 4 ANC visits	59.1 %	[48.4 %–69.0 %]	71.9 %	[64.9 %–78.0 %]	69.3 %	[62.6 %–75.3 %]	64.2 %	[60.2 %–68.1 %]
Counseled for PNC by CHW	6.8 %	[2.3 %–18.6 %]	12.4 %	[7.8 %–19.3 %]	5.9 %	[2.5 %–13.6 %]	3.6 %	[2.4 %–5.5 %]
Districts								
Morogoro DC	6.8 %	[2.2 %–19.4 %]	34.5 %	[13.7 %–63.6 %]	14.8 %	[5.2 %–35.3 %]	10.3 %	[5.2 %–19.5 %]
Mvomero	29.5 %	[13.5 %–52.9 %]	29.3 %	[9.2 %–62.8 %]	33.0 %	[17.1 %–54.0 %]	25.6 %	[16.1 %–38.3 %]
Kilosa	40.9 %	[22.2 %–62.7 %]	29.9 %	[12.0 %–57.1 %]	34.0 %	[20.1 %–51.3 %]	44.5 %	[31.7 %–58.0 %]
Ulanga	22.7 %	[9.7 %–44.7 %]	6.3 %	[1.5 %–23.3 %]	18.2 %	[7.1 %–39.3 %]	19.6 %	[10.9 %–32.5 %]
Distance								
Facility in village	70.5 %	[47.9 %–86.1%]	34.5 %	[14.2 %–62.6 %]	70.9 %	[53.6 %–83.8 %]	62.6 %	[49.4 %–74.2 %]
1 to 5 KM	11.4 %	[3.8 %–29.4 %]	46.6 %	[21.3 %–73.7 %]	8.4 %	[3.2 %–20.0 %]	16.6 %	[9.0 %–28.5 %]
5 or more KM	18.2 %	[6.2 %–42.7 %]	19.0 %	[4.7 %–52.6 %]	20.7 %	[10.2 %–37.5 %]	20.7 %	[12.0 %–33.5 %]

Column percentages are based on non – missing values. Data on place of PNC missing for 59 (3.1 %) of women

### Bivariate analysis

Bivariate analysis (Table 2) showed women were significantly more likely to use a health facility for postnatal care if the mode of delivery was caesarean section or forceps delivery (OR 2.6, 95 % CI 1.65–4.07), there were complications during the intra partum period (1.51, 1.09–2.09) or in the postnatal period (1.56, 1.09–2.22), or the woman received counseling from a CHW about postnatal care (2.58, 1.55–4.29). Significantly, higher odds were also observed if the baby received testing for HIV (1.9, 1.2–3.02) or if partner was tested for HIV (1.35, 1.05–1.74). Lower odds were associated with delivery at a dispensary (0.54, 0.38–0.77). The only community level variable associated with significant odds of receiving postnatal care was the geographic location of the respondent. Women living in Mvomero (0.5, 0.22–1.16) Kilosa (0.33, 0.15–0.71) and Ulanga (0.3, 0.12–0.72) were less likely to receive postnatal care at a facility than those living in Morogoro DC.

**Table 2:** Determinants of use of postnatal care in rural Tanzania; results for multilevel logistic models

Variables		Unadjusted odds ratios			Model 1			Model 2			Model 3		
		(*N* = 1931)			(*N* =1889) (Individual variables)			(*N* = 1909) (Community variables)			(*N* = 1889) (Individual + Community variables)		
		OR	95 % CI	*p* value	OR	95 % CI	*p* value	OR	95 % CI	*p* value	OR	95 % CI	*p* value
Individual level													
Age (in years)	15–19	1.00 (ref)			1.00 (ref)						1.00 (ref)		
	20–34	1.13	0.80, 1.60	0.47	1.04	0.67, 1.62	0.86				1.14	0.64, 1.57	0.99
	35–49	1.31	0.85, 2.01	0.20	1.18	0.68, 2.03	0.56				1.16	0.66, 1.96	0.64
Birth order	First pregnancy	1.00 (ref)			1.00 (ref)						1.00 (ref)		
	Second or higher	1.17	0.87, 1.59	0.30	1.13	0.76, 1.68	0.54				1.13	0.76, 1.68	0.54
Education	Less than primary	1.00 (ref)			1.00 (ref)						1.00 (ref)		
	Primary or higher	1.28	0.99, 1.65	0.06	1.38*	1.05, 1.82	0.03				1.37*	1.04, 1.81	0.03
Wealth quintile	Lowest	1.00 (ref)			1.00 (ref)						1.00 (ref)		
	Lower	0.86	0.58, 1.26	0.43	0.89	0.59, 1.33	0.55				0.86	0.57, 1.29	0.46
	Middle	0.91	0.62, 1.32	0.61	0.89	0.60, 1.32	0.54				0.86	0.58, 1.28	0.45
	Higher	0.96	0.65, 1.4	0.84	1.02	0.68, 1.53	0.93				0.96	0.64, 1.45	0.85
	Highest	0.92	0.61, 1.37	0.67	0.86	0.56, 1.33	0.48				0.79	0.51, 1.23	0.28
Place of Delivery	Home	1.00 (ref)			1.00 (ref)						1.00 (ref)		
	Hospital	0.7	0.51, 1.06	0.10	0.52^	0.35, 0.79	0.002				0.50^	0.33, 0.76	0.001
	Health Center	0.75	0.52, 1.09	0.13	0.64*	0.43, 0.95	0.03				0.57^	0.38, 0.85	0.01
	Dispensary	0.54^	0.38, 0.77	0.01	0.49#	0.34, 0.72	<0.001				0.48#	0.33, 0.69	<0.001
Antenatal Care utilization	No ANC visits	1.00 (ref)			1.00 (ref)						1.00 (ref)		
	1–3 visits	2.15	0.73, 6.32	0.16	2.15	0.72, 6.45	0.17				2.37	0.79, 7.08	0.12
	4+ visits	2.27	0.78, 6.61	0.13	2.50	0.84, 7.43	0.10				2.71	0.91, 8.06	0.07
Mode of delivery	Normal delivery	1.00 (ref)			1.00 (ref)						1.00 (ref)		
	Cesarean/forceps	2.6*	1.65, 4.07	<0.001	2.89#	1.76, 4.73	<0.001				2.95#	1.80, 4.81	<0.001
Seizures/unconscious antenatally	No	1.00 (ref)			1.00 (ref)						1.00 (ref)		
	Yes	2.18	0.93, 5.1	0.72	2.29	0.94, 5.57	0.07				2.33	0.95, 5.66	0.06
Severe antenatal swelling of face/legs	No	1.00 (ref)			1.00 (ref)						1.00 (ref)		
	Yes	0.71	0.49, 1.05	0.08	0.66*	0.44, 0.99	0.05				0.65*	0.43, 0.97	0.04
Complications during delivery	No	1.00 (ref)			1.00 (ref)						1.00 (ref)		
	Yes	1.51*	1.09, 2.09	0.01	1.31	0.89, 1.94	0.17				1.29	0.87, 1.91	0.2
Complications during Postnatal Period	No	1.00 (ref)			1.00 (ref)						1.00 (ref)		
	Yes	1.56*	1.09, 2.22	0.01	1.39	0.91, 2.11	0.13				1.33	0.88, 2.02	0.19
Report spending money on delivery care	No	1.00 (ref)			1.00 (ref)						1.00 (ref)		
	Yes	0.82	0.64, 1.05	0.12	0.77*	0.59, 1.00	0.05				0.78	0.60, 1.01	0.06
Counseled for PNC by CHW	No	1.00 (ref)			1.00 (ref)						1.00 (ref)		
	Yes	2.58^	1.55, 4.29	<0.001	2.32^	1.37, 3.93	0.002				2.3^	1.36, 3.89	0.002
Received HIV testing for infant	No	1.00 (ref)			1.00 (ref)						1.00 (ref)		
	Yes	1.9^	1.2, 3.02	0.01	1.93^	1.19, 3.15	0.01				1.94^	1.19, 3.15	0.01
Partner tested for HIV	No	1.00 (ref)			1.00 (ref)						1.00 (ref)		
	Yes	1.35*	1.05, 1.74	0.02	1.42*	1.08, 1.87	0.01				1.41^	1.07, 1.86	0.01
Community level													
Community usage of family planning	Low	1.00 (ref)						1.00 (ref)			1.00 (ref)		
	Middle	1.54	0.8, 2.96	0.19				1.53	0.82, 2.86	0.18	1.57	0.83, 2.98	0.16
	High	1.82	0.95, 3.5	0.07				2.21*	1.04, 4.69	0,04	2.48*	1.15, 5.37	0.02
Community coverage of 4 ANC visits	Low	1.00 (ref)						1.00 (ref)			1.00 (ref)		
	Middle	0.77	0.4, 1.46	0.42				0.73	0.41, 1.31	0.30	0.70	0.38, 1.27	0.5
	High	1.6	0.85, 3.02	0.15				1.22	0.66, 2.24	0.52	1.14	0.61, 2.14	0.68
Community level of poverty	Low	1.00 (ref)						1.00 (ref)			1.00 (ref)		
	Middle	0.81	0.42, 1.55	0.52				0.89	0.51, 1.56	0.67	1.05	0.59, 1.88	0.89
	High	1.39	0.72, 2.69	0.33				1.16	0.57, 2.38	0.70	1.45	0.68, 3.09	0.34
Community trust	Low	1.00 (ref)						1.00 (ref)			1.00 (ref)		
	High	1.44	0.83, 2.48	0.19				1.76*	1.12, 2.75	0.01	1.77*	1.12, 2.79	0.01
Community level of education	Low	1.00 (ref)						1.00 (ref)			1.00 (ref)		
	Middle	1.0	0.52, 1.93	0.99				0.72	0.39, 1.33	0.29	0.68	0.36, 1.26	0.22
	High	1.49	0.77, 2.89	0.23				1.27	0.66, 2.46	0.47	1.20	0.61, 2.36	0.60
Distance to nearest health facility	Facility in village	1.00 (ref)						1.00 (ref)			1.00 (ref)		
	0–5 KM	1.77	0.87, 3.59	0.11				1.08	0.59, 1.99	0.82	1.07	0.57, 1.99	0.86
	5 or more KM	1.09	0.55, 2.15	0.81				1.39	0.76, 2.55	0.30	1.38	0.74, 2.57	0.32
Districts	Morogoro DC	1.00 (ref)						1.00 (ref)			1.00 (ref)		
	Mvomero	0.50	0.22, 1.16	0.11				0.41*	0.20, 0.85	0.02	0.37*	0.17, 0.79	0.01
	Kilosa	0.33^	0.15, 0.71	0.01				0.31^	0.15, 0.61	<0.001	0.27#	0.13, 0.56	<0.001
	Ulanga	0.3^	0.12, 0.72	0.01				0.16#	0.06, 0.39	<0.001	0.15#	0.06, 0.38	<0.001
Variance					0.48			0.96			0.47		
SE (variance)					0.1			0.12			0.1		

*p < 0.05 ^*p* < 0.01 #*p* < 0.001

### Multilevel analysis

When a multi-level analysis was performed (Table 2), the null model (the multi-level random intercept logistic regression model without covariates), resulted in unexplained variance of 20 % at the level of the clusters. The full model with individual and community level covariates reduced the unexplained variance to 12 % at the cluster level showing that the variation in the use of postnatal care was explained best by the inclusion of both individual and community-level characteristics. The likelihood ratio test was applied to test the significance of the random intercept model versus simpler logistic regression models and was significant (*p* < 0.001) showing that the multilevel model is the better model to explain the use of postnatal care. Models with random slope and interaction variables did not prove significantly different from the random intercept model and are not shown here. The significance of the random intercept in the null model (*p* <0.001) implies that a significant component of the variance in the use of postnatal care was not captured by the observed covariates and these unmeasured variables may constitute a source of bias.

### Individual level effects

Women who had completed primary level of education or higher were more likely to go for postnatal care at a health facility (OR 1.37, 95 % CI 1.04–1.81). Those delivering at a health facility, including at a hospital (0.50, 0.33–0.76), health center (0.57, 0.38–0.85), or dispensary (0.48, 0.33–0.69), were less likely to use postnatal care respectively as compared to those delivering at home. Women reporting swelling of face and legs during pregnancy (*n* = 240) were 35 % less likely to go for postnatal care (0.65, 0.43–0.97). Women who had a complicated mode of delivery (Cesarean section/ forceps delivery) were 2.9 times (2.95, 1.8–4.81) more likely to report receiving postnatal care services from facilities. Women counseled by a CHW on postnatal care were 2.3 times more likely to use a facility for postnatal care (2.3, 1.36–3.89). HIV testing of infants was associated with almost two-fold higher odds of receiving postnatal care at a facility by women (1.94, 1.19–3.15) while women whose partners were tested for HIV also had increased odds (1.41, 1.07–1.86) (Table 2).

### Community level effects

Women living in Mvomero, Kilosa and Ulanga were 59 % (0.37, 0.17–0.79), 69 % (0.27, 0.13–0.56) and 84 % (0.15, 0.06–0.38) less likely, respectively, to access postnatal care than those living in Morogoro DC. Women from communities that had high postpartum contraceptive use prevalence were more likely to access postnatal care (2.48, 1.15–5.37) but not those with high levels of 4 or more antenatal visit coverage. Communities with high level of trust were more likely to use postnatal care than communities with lower levels of trust in the health system on issues related to maternal health (1.77, 1.12–2.79). Community education, poverty levels, and distance to nearest facility (which has been widely used as an indicator of geographic access) did not appear to have any influence on utilization of postnatal care (Table 2).

## Discussion

As emphasized in the beginning, our study is focused on the points of contact for postnatal care for the mother, which is delivered only at health facilities only in the Tanzanian context. A strongpoint of our study is the inclusion of community level factors that may affect the use of postnatal care at health facilities, and indeed, reduced the unexplained variance at the cluster level. Use of postnatal care is low in rural Morogoro in Tanzania with less than one in four women reporting having visited a health facility for postnatal care. This is lower than the DHS estimate for Tanzania and Morogoro region from 2010 (35.4 %), and explained by the fact that our sample excluded large urban centers, which tend to have better services [4]. The lower estimate also is reflective of the exclusion of any postnatal care delivered as part of pre-discharge care after child birth at a health facility.

Among the predisposing characteristics, maternal education, geographic place of residence and communities with high postpartum contraceptive prevalence were associated with use of PNC. In our study, women’s education had a strong association with use of postnatal care at facilities similar to the findings by Fort et al. from DHS surveys in Rwanda [23]. Higher educational attainment is more often associated with higher socio-economic status and greater awareness of maternal health care services [24]. Education could help women attain greater autonomy and capable of negotiating and interacting with their family and community to access services and educated communities are capable of demanding better public services. Morogoro DC was associated with a significantly higher likelihood of women accessing postnatal care than the other study districts in the region (Mvomero, Kilosa and Ulanga). Morogoro DC is situated very close to a major town with a large regional hospital and multiple donor programs in maternal health, resulting in better access. The association with levels of postpartum family planning in the community may be a proxy for the health behaviors and community norms as well as quality of health services available to the community [24]. This acts to shape their intentions to seek postnatal care according to the Andersen model of health care utilization [20].

Other factors such as mother’s age at delivery, birth order, and wealth were not significant predictors of use of postnatal care in our study but have been found significant predictors elsewhere [25–27]. Spending money to access care was associated with lower likelihood of postnatal care when individual level factors alone are considered but the significance disappeared when community level factors were added to the model. Other studies have emphasized the importance of cost as a barrier to the use of postnatal health services in Low & Middle Income Countries (LMIC) with one study from Tanzania reporting that 45% of women do not have a cash income and that the financial costs of accessing health services, can be beyond the ability of families to pay [28–30]. Our finding that distance was not associated with facility use for postnatal care unlike other studies [31–33] may be because Morogoro has a relatively better availability of health facilities with 73 % of women sampled living within 5 km of a health facility and 61 % living in a village with a facility.

Among the enabling characteristics, counseling from a CHW, partner testing for HIV status and trust in health system were associated with PNC use. Women counseled by a CHW to go to a facility for postnatal care were more likely to use postnatal services. CHW coverage in the region at the time of the baseline survey was low, and CHW did not routinely provide MNCH services. This reflects the need for building capacity among CHWs to counsel women to seek facility based postnatal services at the community level in addition to strengthening the services at the level of facilities. Male participation in care of the antenatal woman has been shown to have a positive effect on the acceptance of perinatal interventions [34]. Partner testing for HIV increasing use of PNC may be reflective of this increased acceptance of health interventions. Living in communities where women trust the health system on matters related to pregnancy and childbirth significantly increased the likelihood of seeking postnatal care. Gilson et al. propose two components of trust in the context of patient provider relationship - inter-personal trust and institutional trust [35]. However, the present analysis only explores the component of interpersonal trust that may be shaped by repeated interactions during the pregnancy. Repeated contact with health workers during pregnancy through antenatal care services could promote confidence and familiarity with the health system leading to increased trust in the health system.

Need characteristics like HIV testing for baby, antenatal complication of severe swelling of face and legs, mode of delivery and place of delivery care were also associated with use of PNC. HIV testing of the infant is offered only to women who test positive during pregnancy or delivery. This indicates that women who are likely to be seropositive for HIV and require test for their newborn are much more likely to seek PNC. Those with a complicated mode of delivery like a cesarean section or forceps delivery are more likely to come for postnatal services as part of follow up care for their complication. Women reporting severe swelling of face and legs as a symptom (possibility of severe anemia) in the antenatal period, were significantly less likely to use postnatal care but we were unable to observe similar associations with reporting of other antenatal complications including eclampsia (*n* = 29) due to small numbers in our sample. These women may have been unable to walk to health facilities even if they were living near one. This gap in knowledge warrants further research to validate and fully understand the relationships between anemia and other antenatal complications and use of postnatal care. Although complications during the intra-partum period or postnatal period were observed to be significantly associated with PNC in the bivariate analysis, they failed to achieve significance in the final model, possibly, due to their low prevalence in the sample.

Women delivering at home were significantly more likely to visit a health facility for postnatal care than those who delivered at a hospital, health center or dispensary. In previous studies, women receiving delivery care at a facility were considered, by default, as receiving postnatal care. In our study, we have considered only women who made a visit separate from the visit for delivery as having made a postnatal visit. This difference in the definition of what is considered a postnatal care visit between our study and previous research may be a reason for some of the differences in findings. It is possible that women delivering at facilities were not being advised to return for care visits while women delivering at home feel the need to be checked or register the births by making a visit to a facility. Qualitative research conducted during the same time period as our survey suggests that women come to the facility in the days after delivery with the specific intention of getting a card for the baby required for immunization and other services and end up receiving postnatal care (personal communication).

### Limitations

We have interpreted postnatal care as any care sought for the mother at a health facility within 6 weeks of delivery, irrespective of the reason for seeking care (routine care vs. care seeking for complications). With our broad entry criteria, our strategy has the possibility of introducing non-systematic bias, which will reduce the magnitude of association between the outcome and predictors. Other limitations to this study include the small sample size of the level 2 groups (60 clusters) and the low precision for group-level variables resulting in wide confidence intervals. The villages in the Morogoro region of Tanzania vary greatly in size and structure. Sub-villages (or Vitongoji) may be so far from others of the same village unit that it may be difficult to group them as one homogenous cluster. Lack of accurate data on visits to health facilities in terms of time taken, precise costs incurred on transport and difficult terrain may hinder the precise measurement of geographic and economic access. Other than district of geographic residence, all the community variables were constructed by aggregating the individual-level characteristics at the community level.

The significance of the random intercept in the null multilevel model shows that many variables were not be accounted for in the multilevel analysis, particularly the different programmatic activities carried out at different levels of the health system or the targeted selection of communities into health system strengthening programs and projects. Traditional beliefs about the postnatal period play an important part in the use of care and were not adequately captured in our household survey. The analysis could not address the effects of these unobserved or unmeasured variables due to the lack of appropriate explanatory variables. Investigation of the quality of services provided was outside the scope of this study. Moreover, in a cross-sectional survey, it is not possible to determine direction of causality.

### Policy implications

Rural Morogoro is comparable to rural communities in other low resource settings with low postnatal care. At the district level, it would be helpful to look at the variations in use of postnatal care and critically analyze the factors for the differences. The differences in postnatal care use associated with place of delivery merit a more detailed investigation. Another key programmatic area of enquiry is the need to strengthen the counseling of women about the postnatal period and the benefits of returning to the facilities for postnatal care services.

## Conclusions

Postnatal care (not including pre-discharge care) use is universally low in Morogoro with significant geographic differences. The study findings represent a step toward an improved understanding of factors influencing women’s use of postnatal care in Tanzania and other maternal health services globally. Measurement and understanding of individual and community level factors, allow for better targeting and selection of communities for health system strengthening activities. This study reinforces the need to understand the contextual complexities of care seeking and the importance of building and strengthening systems through linkages between facility and community.
